# Microbiological profiles of fungal keratitis: a 10-year study at a tertiary referral center

**DOI:** 10.1186/s12348-016-0071-6

**Published:** 2016-02-20

**Authors:** Joanne W. Ho, Mark M. Fernandez, Rachelle A. Rebong, Alan N. Carlson, Terry Kim, Natalie A. Afshari

**Affiliations:** Shiley Eye Institute, University of California San Diego, San Diego, CA USA; Duke Eye Center, Duke University School of Medicine, Duke University Medical Center, Durham, NC USA; Shiley Eye Institute, Department of Ophthalmology, University of California San Diego, 9415 Campus Point Dr. #0946, La Jolla, CA 92093-0946 USA

**Keywords:** Fungal keratitis, Cornea, Microbiology, Contact lens, Penetrating keratoplasty

## Abstract

**Background:**

Given the rise in cases of fungal keratitis in recent years, this study was performed to better elucidate the microbiological profile, risk factors, and surgical intervention rates of fungal keratitis at a tertiary referral center in the Southeastern USA.

**Findings:**

This is a retrospective case series of fungal keratitis infections treated at Duke University Eye Center from January 1, 1998, to October 6, 2008. Of the 4651 culture-proven corneal ulcers identified, 63 (1.4 %) were positive for fungal keratitis with a total of 69 fungal organisms isolated. The majority of isolates were filamentous species (44 of 69, 64 %), and the most commonly isolated organism was *Curvularia* (11 of 69, 16 %). Bacterial coinfections were found in 24 of the 63 cases (38 %). The most commonly associated risk factors were contact lens wear (*n* = 15, 24 %) and prior penetrating keratoplasty (PKP) (*n* = 15, 24 %). Twenty-three cases (37 %) required surgical intervention. The rate of surgical intervention was highest in patients with prior PKP (7/15, 47 %).

**Conclusions:**

In this study, the leading risk factors for fungal keratitis were contact lens wear and prior PKP. Filamentous species were the most common causative pathogens. A relatively high rate of mixed bacterial-fungal infections was found. Patients with prior PKP were more likely to require surgery than patients without history of keratoplasties.

## Findings

### Introduction

Fungal keratitis is an important cause of ocular morbidity and blindness worldwide [1]. Although the condition is still relatively uncommon in the USA, recent studies have shown an increasing number of cases in both contact lens wearers and non-contact lens wearers at multiple centers across the country [2]. Additionally, the incidence and microbiological profile of fungal keratitis vary widely depending on geographic location [1, 3, 4]. The infection is rare in temperate areas and more common in warm and humid environments [3, 5]. Given these factors, it is important to establish ongoing surveillance in different geographic regions to monitor cases of fungal keratitis.

### Methods

With institutional review board (IRB) approval, we conducted a database search for all patients treated for culture-positive infectious keratitis between January 1, 1997, and October 6, 2008, at Duke University Eye Center. Each patient was started on antifungal treatment within the day of initial presentation. Only cultures found positive for corneal fungal growth and fungal isolates were included in this study. The media used for speciation was inhibitory mold agar with gentamicin and chloramphenicol. There were no criteria for exclusion. The following risk factors were recorded for each case: contact lens wear, past corneal surgery, human immunodeficiency virus (HIV) status, diabetes status, history of herpes simplex virus (HSV) infection or other causes of neurotrophic cornea, other ocular surface disease, exposure keratopathy, and recent vegetable matter injury to the eye. Additional data recorded included patient age, patient gender, fungal speciation, and surgical intervention, if performed. Presence and speciation of any bacterial coinfection was also recorded.

### Results

A total of 4651 culture-proven corneal ulcers were identified in the study period. Sixty-three ulcers in 63 patients were culture positive for fungus (1.4 %). The mean age of patients was 56.1 years (range 0.5 to 89 years). Thirty-four patients (54 %) were men and 29 (46 %) were women. Documented predisposing risk factors were present in 51 of the 63 patients (81 %). The most common risk factors were contact lens wear (15 eyes, 24 %) and prior penetrating keratoplasty (PKP) (15 eyes, 24 %). A total of 10 cases (16 %) were found in patients with diabetes, and 9 eyes (14 %) suffered recent traumatic corneal injury with vegetable matter. Additional risk factors (Table 1) included bandage contact lens wear (four eyes, 6.3 %), prior HSV neurotrophic ulcers without history of PKP (four eyes, 6.3 %), other ocular surface diseases (Stevens-Johnson syndrome, ocular cicatricial pemphigoid, and symblepharon) (three eyes, 4.8 %), and recent history of exposure keratopathy (one eye, 1.6 %). One patient (1.6 %) was HIV positive, and one patient developed fungal keratitis after suffering head trauma requiring hospitalization. No eye had a history of corneal refractive surgery.

**Table 1:** Associated conditions in cases of fungal keratitis

Risk factor	No. eyes/cases (*N* = 63)	Percentage
Refractive contact lens	15	24
Previous penetrating keratoplasty	15	24
Unknown risk factors	12	19
Diabetes	10	16
Traumatic injury with vegetable matter	9	14
Bandage contact lenses	4	6.3
Herpes simplex keratitis	4	6.3
Human immunodeficiency virus positive	1	1.6
Head trauma	1	1.6
Ocular Stevens-Johnson syndrome	1	1.6
Ocular cicatricial pemphigoid	1	1.6
Symblepharon	1	1.6
Exposure keratopathy	1	1.6

Percents do not add to 100 % because 11 of 64 cases had >1 risk factor

Forty-four of the 69 isolates cultured (64 %) were filamentous forms, 22 (32 %) were yeast forms, and 3 were recorded as unidentified molds (4.3 %). *Curvularia* (*N* = 11), *Fusarium* species (*N* = 10), and *Aspergillus* species (*N* = 10) were the most common filamentous isolates (Table 2). The *Candida* species represented all of the speciated yeasts except one, which was an unidentified yeast form. One eye that had suffered corneal trauma with vegetable matter grew four different species (*Fusarium dimerum*, a species of *Curvularia*, *Aspergillus fumigatus*, and *Aspergillus niger*). Cultures from two of the eyes each grew two species of *Candida*. One case speciated both unidentified molds and yeasts.

**Table 2:** Pathogenic organisms identified in cases of fungal keratitis

Organism	No. isolates (*N* = 69)	Percentage
Filamentary species	44	64
*Curvularia*	11	16
*Fusarium*	10^a^	14
*Aspergillus*	10	14
*Paecilomyces*	5	7.2
*Cladosporium*	2	2.9
*Cryptococcus*	1	1.4
*Bipolaris*	1	1.4
*Scytalidium*	1	1.4
*Colletotrichum*	1	1.4
*Alternaria*	1	1.4
*Epicoccum*	1	1.4
Unidentified molds	3^b^	4.3
Yeast species	22	32
*Candida albicans*	9^c^	13
*Candida parapsilosis*	6	8.7
*Candida glabrata*	2	2.9
*Candida tropicalis*	2	2.9
*Candida krusei*	1	1.4
*Candida guilliermondii*	1	1.4
Unidentified yeasts	1	1.4

^a^One case grew *Fusarium dimerum*, a species of *Curvularia*, *Aspergillus fumigatus*, and *Aspergillus niger*

^b^One case grew both unidentified mold and yeast species

^c^One case grew both *C. albicans* and *C. parapsilosis*. Another case grew both *C. albicans* and *C. tropicalis*

Twenty-four of the 63 eyes (38 %) had mixed bacterial-fungal infections. *Coagulase-negative staphylococcus* was the most common coinfection, affecting 10 eyes (4 with *Curvularia*, 2 with *Aspergillus*, 1 with *Candida*, 1 with *Fusarium*, 1 with *Scytalidium*, and 1 with both *Pseudomonas* and *Candida*). The next most common bacteria found were *Propionibacterium* (present in five cases) and *Pseudomonas* (present in two cases). The remaining cases of coinfections involved *Staphylococcus aureus*, *Serratia*, *non-hemolytic Streptococcus*, *Corynebacterium*, and mixed Gram-positive and negative flora.

In the entire case series, 23 of the 63 eyes (37 %) required surgical intervention. Twenty eyes (20/63, 32 %) underwent therapeutic PKP as initial surgical therapy. Nine keratoplasties were performed for persistence of keratitis after medical therapy, five were indicated because of impending or frank corneal perforation, five were repeat keratoplasties for failed prior PKPs due to fungal keratitis, and one was for recurrent fungal keratitis. Two eyes were enucleated (2/63, 3.2 %), and one was eviscerated (1/63, 1.6 %) in the acute stage for overwhelming infection.

Seven of the 15 eyes (47 %) with prior PKP required surgical interventions, of which 5 were repeat PKPs, 1 was an evisceration, and 1 was an enucleation. Of eyes with contact lens-associated fungal keratitis without previous PKP (14 of the 15 contact lens wearers), 36 % (*N* = 5) received surgical intervention (all PKPs).

### Discussion

Infectious keratitis remains an important cause of corneal ulcers in the USA. Studies have shown variable trends in risk factors, microbial profiles, and surgical outcomes of fungal keratitis [2, 6–10]. In our study, contact lens wear was one of the leading risk factors (24 % of cases) but at a significantly lower rate than that found in recent multicenter studies—41 % reported by Gower et al. [2] and 37 % reported by Keay et al. [6]. This may simply represent a lower incidence of contact lens use among our patient population or a higher relative incidence of other predisposing factors for fungal keratitis. For instance, previous PKP was present in just as many cases associated with contact lens wear (24 % of cases) in our series. This represents a change from the findings of the Keay et al. study, in which prior PKP was a much less significant risk factor (65/733, 8.9 %). Similarly, diabetes mellitus was a much more prevalent risk factor in our study (10/64, 16 %) as compared to results in the most recent study from South Florida [7] (6/84, 7.1 %).

The spectrum of pathogens isolated was not unexpected given the climate of the study area. North Carolina and most of the Southeastern USA have a humid, subtropical climate—warmer and more humid than the Northeast, where *Candida* predominates, but cooler than South Florida, where *Fusarium* accounts for most cases [7–9, 11, 12]. As might be expected, the majority of pathogenic organisms in this study were filamentary species (64 %): predominantly *Curvularia*, *Fusarium*, and *Aspergillus*.

We also found a high prevalence of mixed bacterial-fungal corneal infections, representing 24 of 63 cases (38 %). This is significantly higher than the prevalence of bacterial-fungal infections reported in the Northeastern USA (11/61, 18 %) [8]. This increased rate of polymicrobial infections may reflect the high number of patients with prior PKP in our series as these patients were likely more susceptible to superinfections.

The overall surgical intervention rate found in our study (37 %) is significantly higher than the 26 % of cases reported in a recent multicenter study [6]. This could be attributable to the high rate of prior PKP found as a risk factor in our study as the rate of surgical intervention required was much higher in patients with prior PKP (47 %) than in contact lens wearers without prior PKP (36 %). The increased likelihood of requiring surgical intervention in eyes with prior PKP may reflect a higher severity of disease at presentation for a number of reasons. First, a corneal graft is essentially neurotrophic, masking the early symptoms and signs of infection. Second, the use of chronic steroids decreases the immune response to infection. Third, the presence of other pathology like corneal scarring or stromal disease can make early infections more difficult to detect. Finally, eyes with prior PKP have corneal wounds and suture tracks that provide easier access to the anterior chamber of the eye, making it more vulnerable to endophthalmitis.

## Abbreviations

HIV: human immunodeficiency virus
HSV: herpes simplex virus
PKP: penetrating keratoplasty
